# The Rearing and Biology of the Desert Beetle, *Microdera punctipennis*, Under Laboratory Conditions

**DOI:** 10.1673/031.011.0139

**Published:** 2011-03-30

**Authors:** Yan Wang, Xiaoning Liu, Jia Zhao, Kelaimu Rexili, Ji Ma

**Affiliations:** Xinjiang Key Laboratory of Biological Resources and Genetic Engineering, College of Life Science and Technology, Xinjiang University, 14 Shengli Road, 830046 Urumqi, China

**Keywords:** Central Asia, developmental duration, immature stages, Tenebrionidae

## Abstract

*Microdera punctipennis* Kasz (Coleoptera: Tenebrionidae) is a unique species that lives in the desert region of Central Asia and has adopted a nocturnal habit to survive the desert environment. Female adults are larger in size than male adults. The female/male ratio was 1.04:1. A rearing method using reused plastic bottles was used. The rearing conditions were 30 ± 0.5°C, 30 ± 6% relative humidity (RH), and 16:8 L:D photoperiod. Cabbage was provided as food. Cannibalism was avoided by rearing one larva in a bottle. A complete life cycle was obtained under these conditions. The viability of eggs, larvae, prepupae, pupae, and teneral adults was 93.54%, 83.71%, 84.76%, 87.64%, and 93.59%, respectively. Embryogenesis took 7.35 days on average. The larval duration in each instar was 2.25 days. The mean duration of the larvae, prepupae, pupae, and teneral adult was 49.27, 7.05, 9.95, and 10.12 days, respectively. The coloration of each developmental stage gradually changed from creamy white to light brownish or black. Females commenced oviposition when their body color became black. On average, each female produced 568 eggs.

## Introduction

Beetles, especially Tenebrionidae, are among the most successful animals of the desert, and are called “indicators of desertization” (Ren and Yu 1999). They adopt several strategies to survive hostile environments. Behaviorally and morphologically the majority of desert beetles are active at night. During the day they bury themselves deeply in the substrate to avoid high temperatures and low humidity (Wharton 1983; Cloudsley-Thompson 1990) and take up fog-water as a water source (Seely 1979; Parker and Lawrence 2001; Hamilton et al. 2003; Adam 2004). They tend to have a flattened body and short legs, meaning they are well adapted to burrowing in sand. Body size is an important feature for adaptation to microclimate and substrate factors (Thomas 1983; Doyen and Slobodchikoff 1984). Certain structural and physiological regulations developed by the desert beetles also play an important role in desert adaptation. For instance, the subelytral cavity, an airtight space formed by the fusion of the elytra (Draney 1993; Gorb 1998), is found especially in desert Tenebrionidae (Cloudsley-Thompson 2001) and it helps to lower cuticular water permeability in desert beetles (Zachariassen 1991, 1996). Desert tenebrionid beetles also adopt seasonal behavioral changes to avoid hostile conditions; most species exhibited 7–10 month of activity with one or two peaks of abundance (Krasnov and Ayal 1995).

*Microdera punctipennis* Kasz (Coleoptera: Tenebrionidae), is a small, flightless beetle adapted to live in the Gurbantonggut desert (Huang et al. 2005), the second largest desert in northwest China. Antifreeze protein genes from *M. punctipennis* have been cloned (Zhao et al. 2005) and functionally characterized (Wang et al. 2008; Qiu et al. 2010), but little else is known of its biology and immature stages.

This article aims to establish: (i) the morphological and behavioral features of *M. punctipennis* adults collected from its natural environment; (ii) a rearing method for *M. punctipennis* so as to identify the immature stages of *M. punctipennis* under rearing conditions; and (iii) the biological parameters of development of *M. punctipennis* under rearing conditions.

## Materials and Methods

### Adult collection site and observation

*Microdera punctipennis* adults were collected in 2008 from Fukang (44° 24′ N, 087° 51′E, 444 m), which is about 100 km northeast of the geological center of Asia. The annual average air temperature is 5–7.5° C. The highest air temperature is more than 40° C and the lowest is lower than -40° C (Wei and Liu 2000; Qian et al. 2004). Body parameters including cephalic capsule width, pronotum width, elytra width, and body length were measured by vernier calipers. Body weight was measured on a fine electronic scale (AL104, Mettler Toledo).

### Egg collection and observation

Overwintered adults collected in March were used for mating and oviposition. Male and female adults were distinguished when they were mating. One hundred thirteen pairs of field-collected adults in total were reared at uncontrolled laboratory conditions in a plastic container (40 cm length × 24 cm width × 12 cm depth) containing 5 cm deep of dry sand, fed with fresh cabbage. Eggs were collected by sifting the sand to separate eggs from the sand. Egg numbers were counted daily.

Eggs were placed in Petri dishes (15 cm diameter) at 30° C, and hatching time and hatched eggs were daily recorded. To measure egg weight, 50 eggs were weighed in total using an electronic balance (exact within ± 0.1 μg). Before being weighed, eggs were cleaned individually with a drop of distilled water to remove attached sand. The cleaned eggs were observed under an optical stereomicroscope or scanning electronic microscopic (LEO1430VP, LEO, www.zeiss.com). Egg length and width were measured under stereomicroscope equipped with Elements 3.0 software (Nikon SMZ-800, www.nikon.com), and calibrated by an objective micrometer.

### Larval rearing and duration

Discarded plastic bottles (600 ml) were cut off at 4 cm from the mouth. 70ml of water was first added, then 800g of sand, to form a wet sand gradient. Newly hatched 3^rd^ instar larvae were singly placed in the set-up bottle for rearing. Total weight of the rearing bottle was measured, and the water loss was supplied at about 20 day's intervals by syringing from the bottom of the bottle. Larval rearing was maintained at 30±0.5° C, 30±6% RH and 16:8 L:D photoperiod conditions. Larval molting was daily checked to record the duration of each instar, indicated by the molts. The instar number was also determined based on the frequency distribution of body parameters, including larval cephalic capsule width and length, pronotum width and length, body length, and weight. To observe and measure larvae, they were first chilled on ice for 3 minutes, and then photographed and measured under stereomicroscope. The measured larvae were no longer recorded for the developmental duration.

**Table 1.**
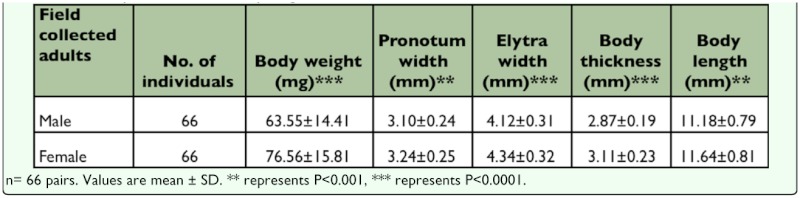
The body dimensions and body weight of field collected adults.

### Prepupal, pupal, and teneral period

Prepupa and pupa were respectively kept in Petri dishes (15cm diameter) under the rearing conditions; the number of pupa was daily recorded. Prepupal body length, body weight, and body thickness of the 8^th^ body segment were measured. Pupal cephalic capsule width, pronotum width and length, body width and length, body weight, and urogomphi length were measured. The teneral period refers to the length of days that the newly emerged adult stays inactive in sand. Teneral cephalic capsule width, pronotum width, elytral width, body length, and weight were measured.

### Statistical analysis

The relationship between larval instars and developmental durations were analyzed by linear regression. Adult body parameters were submitted to student's t-test at two tailed with 5% significance level. The analysis was conducted by GraphPad Prism 4 software.

**Figure 1.**
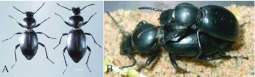
Male and female adults of *Microdera punctipennis.* (A) dorsal view of male (left) and female (right); (B) mating; bar represents 2 mm. High quality figures are available online.

**Table 2.**
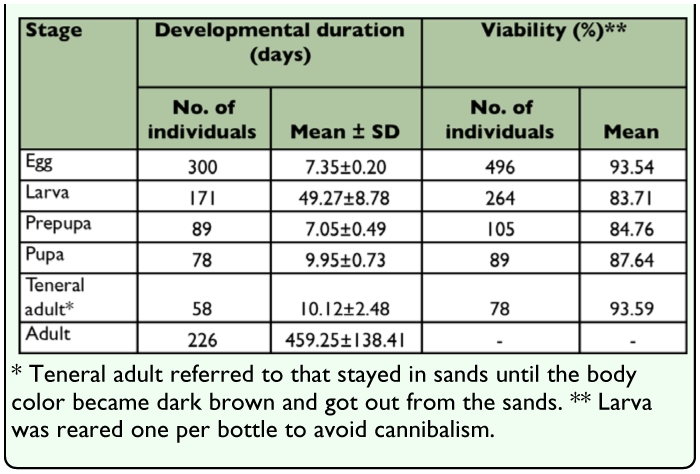
Viability and developmental duration of *M. punctipennis* reared at 30 ± 0.5°C, 30±6% RH, 16L: 8D.

## Results

### *Microdera punctipennis* adult

Mature *M. punctipennis* adults collected from their natural environment were black and small (Figure 1A). Table 1 shows the body dimensions and body weight of field collected adults (n = 66pairs). The body length difference between the male and female was statistically significant (P<0.05), so were the other body parameters (body length, t_130_ = 3.315, P = 0.0012; pronotum width, t_130_ = 3.149, P = 0.0020; elytral width, t_130_ = 4.943, P < 0.0001; body thickness, t_130_ = 4.032, P < 0.0001; body weight, t_130_ = 6.450, P < 0.0001). The ratio of females to males was 1.04:1 (n = 430).

The desert beetle *M. punctipennis* is night active; it feeds, mates, and oviposits at night. Under laboratory conditions, adults were found to feed on immature individuals, damaged eggs, and adults, but not intact eggs. On encountering danger, it contracted its legs, straightened its antennae, and pretended to be dead. For courtship, the male mounted on the female from behind (Figure 1B). Courtship lasted 2–10 minutes, and mating lasted 1–3 minutes.

### Oviposition and egg stages

**Figure 2.**
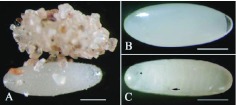
*Microdera punctipennis* egg. (A) natural view of newly laid egg with sand covered; (B) 12-hour-old egg washed with water, an air chamber are seen at one end; (C) 7-day-old egg - short arrow indicates the ochre ocelli, long arrow indicates the tarsungulus of the prothoracic legs of the embryonic larva; bars represent 2mm. High quality figures are available online.

Newly emerged adults began to lay eggs about 20 days after emergence until the end of their lifetime. When laying eggs females inserted the end of their abdomen into the sand, the angle between the body axis and the sand surface was about 20°. After oviposition the female withdrew its ovipositor, kicking sand with its postpedes to bury the eggs. Eggs were laid individually in sand, 3.05 ±1.16 eggs per day, ranging from 1 to 6 (n = 113 pairs). The average number of eggs laid in the first year was 455 per female, and in the second year was 113. The average mass of 50 eggs was 21.70 ± 0.48 mg, and varied from 20.90 to 22.70 mg (n = 400). The egg stage lasted 7.35 ± 0.18 days, varied from 6 to 8 days (n=400). The percentage egg hatchability under the rearing conditions was 93.54% (n = 496) (Table 2).

The newly laid egg was elliptic, creamy white, very delicate, and sticky with sand often attached (Figure 2A). The eggshell became hard and one or two air-chambers within the egg formed after 10 hours (Figure 2B). After 6–7 days, the egg became obtuse and yellowish in appearance. Prior to hatching, red-brown tarsungulus of prothoracic legs and ochre ocelli of the developed larva were visible under the eggshell under the stereomicroscope (Figure 2C). The eggshell surface was homogeneous and compact under scanning electronic microscope.

**Figure 3.**
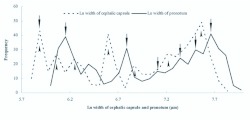
Frequency distributions of logarithmed cephalic capsule width and pronotum width during the larval stages of *Microdera punctipennis.* The most frequently occurring sizes (arrows) of cephalic capsules width and pronotum width identified 7 instars. High quality figures are available online.

### Larval stages

Seven instars in total were determined in *M. punctipennis* by the number of molts as shown in Figure 3. Frequency distributions of larval body parameters, except cephalic capsule length, displayed seven frequency peaks indicating seven instars. Developmental duration (in days) and viability of each larval instar are shown in Table 3. The relative low viability from the 3^rd^ to 5^th^ instars was due to the cannibalism when dozens of larvae were reared per bottle early in this investigation.

The larval body in all instars, except the newly emerged 1^st^ instar and prepupa, was flat and elongated. Body parameters and body weight of each instar are shown in Table 4. Except cephalic capsule length, which showed slow growth rate, all the other body parameters showed a similar growth pattern. Moreover, larval body weight increased very slowly in the first four instars, but dramatically increased in the later instars. Under the rearing conditions, the duration time in each instar proceeded in a linear way. The linear function was Y = 2.25x - 0.83 (R^2^ = 0.97), indicating that the developmental time in each successive instar was 2.25 days longer than in the previous instar. The overall larval stage from the 1^st^ to the 7^th^ instar lasted about 56 days and ranged from 36-78 days (n = 71).

**Table 3.**
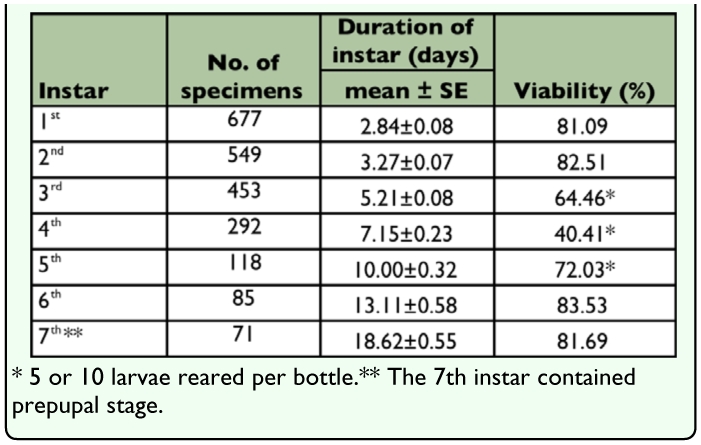
Developmental duration and viability of *M. punctipennis* larval instars reared at 30±0.5°C, 30± 6% RH and 16L:8D.

**Table 4.**
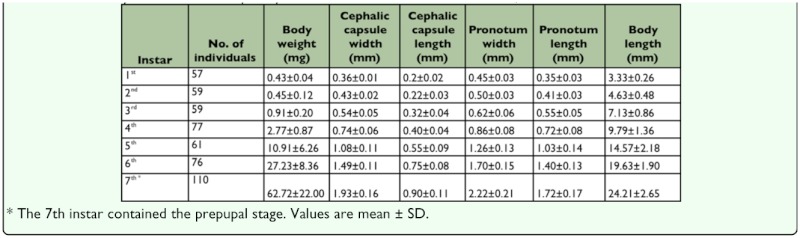
The body dimensions of *M. punctipennis* larval instars reared at 30±0.5°C,30± 6% RH and 16L:8D.

**Figure 4.**
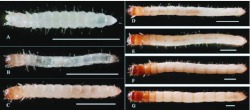
Larval stages of *Microdera punctipennis.* (A) 1^st^ instar; (B) 2^nd^ instar; (C) 3^rd^ instar; (D) 4^th^ instar; (E) 5^th^ instar; (F) 6^th^ instar; (G) 7^th^ instar. Bar represents 2 mm. High quality figures are available online.

Larval color from the 1^st^ to the 7^th^ instar changed gradually from creamy white to yellowish. Head capsule and thorax color changed from creamy white to brown. Meanwhile, the body wall became hard. The 1^st^ instar was delicate; body segments beads as in Figure 4A. The 2^nd^ instar became elongated and transparent; the alimentary tract was visible under a stereomicroscope (Figure 4B).

The 3^rd^ instar was opaque, the head capsule was orange, and a cuplike pigmentation on the pronotum could be viewed (Figure 4C) which gradually intensified in the 4^th^ and 5^th^ instar (Figure 4D, 4E). The 6^th^ instar had two bands on the dorsal side of each abdomen segment; and the cuplike pigmentation disappeared (Figure 4F). The 7^th^ instar was similar with the 6^th^ instar, but much stronger (Figure 4G).

### Larval molting

**Figure 5.**
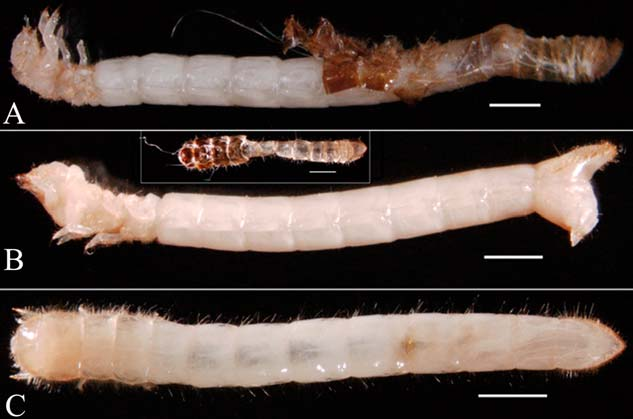
Eclosion of *Microdera punctipennis* larvae. (A) head and thorax emerged first with the help of pygopodium (lateral view); (B) lateral view of the just molted larva with its thorax puckering up and the molted cuticle still on (inset); (C) dorsal view of the larva 10 minutes after molting; bar represents 2 mm. High quality figures are available online.

The first instar emerged either from the head or pygidium. It molted to the 2^nd^ instar without food, but 80% of the starved 2^nd^ instar larvae (n=50) died in the process of exuviation to the 3^rd^ instar. During exuviation the skin split first along the tergal suture of the head and thorax, and then the thorax, head, legs, and abdomen emerged (Figures 5A). This process lasted for about 10 minutes. The newly emerged larva puckered up at the thorax (Figure 5B) and kept inactive for a few minutes. After the thorax became flattened (Figure 5C), larva burrowed into sand. Cannibalism was observed in larvae older than the 2^nD^ instars.

**Figure 6.**
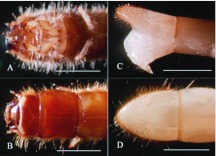
Details of body morphology of *Microdera punctipennis* larva for sand living. (A) sharp and hard tips of prothoracic legs used to excavate sand (ventral view); (B) strongly chitinized labrum, cephalic capsule and pronotum used to thrust sand (dorsal view); (C) strong and sharp pygopods used as center of effort to draw back or molt (lateral view); (D) ossified spines on the last body segment (dorsal view); bar represents 2 mm. High quality figures are available online.

### Larval structures for sand living

Under the rearing conditions, the larva lived in the boundary between dry and wet sand and was active on the surface in dark. Larval prothoracic legs were larger and stronger than the other legs (Figure 6A) and the cephalic capsule was hard (Figure 6B). These structures together, with their two pygopods and ossified spines on the last body segments (Figures 6C, 6D), help the larva tunnel into sand. Upon pupation, the full-grown larva burrowed a hole in the wet sand for pupation.

### Prepupa and pupa stages

**Figure 7.**
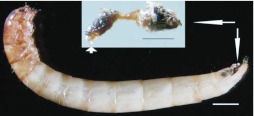
Prepupa of *Microdera punctipennis* with its anus blinded with meconium (lateral view). Bar represents 2 mm. Inset shows the dissected meconium (bar represents 1 mm), short arrow indicates the part inside body and long arrow indicates the part outside body. High quality figures are available online.

**Figure 8.**
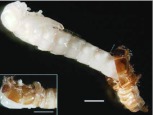
Pupating of *Microdera punctipennis.* Inset shows the initial stage. Bars represent 2 mm. High quality figures are available online.

In the prepupal stage, the body was cylindrical L-shaped, yellowish, and motionless (Figure 7). Pygopods withdrew and attached to the tergite. The anus was plugged with solid meconium. Exuviation also started when the skin split along the tergal suture of the head and thorax (Figure 8). Body parameters of the prepupa are showed in Table 5. The prepupal period lasted 7.05 ± 0.49 days, ranged from 6–8 days, and viability was 84.76% (n = 105).

**Figure 9.**
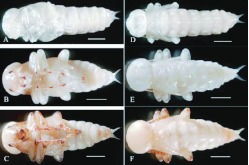
Color change of *Microdera punctipennis* pupa. (A) ventral view of a just emerged pupa; (B) ventral view of a 5-day pupa; (C) ventral view of a pre-molt pupa; (D) dorsal view of a just emerged pupa; (E) dorsal view of a 2-hour pupa; (F) dorsal view of a pre-molt pupa; bars represent 2 mm. High quality figures are available online.

The newly emerged pupa (Figure 9A) was totally creamy white and semitransparent. It folded all its appendages on the ventral surface. A pair of antennae and prominent eyes appeared. Two concavities on the pronotum of the newly emerged pupa were observed (Figure 9D), but disappeared about 2 hours later (Figure 9E). Two days after emergence the color of the eyes, antennae, mandibles, and claws gradually changed from white to black (completely black on day 7) (Figure 9B, 9C). Antenna segmentation was visible at this time (Figures 9C, 9F). Pupal duration lasted 9.95 ± 0.73 days, ranged from 8–12 days, and viability was 87.64% (n = 89). Compared with prepupa, pupal body weight and length were decreased, but **body** width increased (Table 5).

### Teneral adult stages

**Figure 10.**
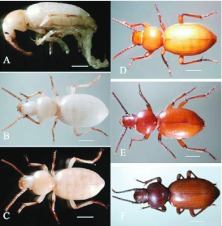
Teneral adult of *Microdera punctipennis.* (A) lateral view of an adult emerging from its pupa with pupal case attached; (B) dorsal view of a just emerged adult; (C) dorsal view of an one-hour adult; (D) dorsal view of an one-day adult; (E) dorsal view of a 3-day adult; (F) dorsal view of a 7-day adult; bars represent 2 mm. High quality figures are available online.

Adult molting occurred at night. The newly emerged adult was creamy white, except the mandibles, tarsal claws, and the joints of antennae and legs (Figures 10A, 10B). During the teneral stage, body color changed progressively from white to black (Figure 10D, 10E, 10F); meanwhile, the elytra and body wall became hard. The newly emerged terneral adult was very delicate with wrinkled elytra (Figure 10B). One hour later, the wrinkled elytra became smooth (Figure 10C), and elytral veins were visible in the dorsal view. The inactive adult stayed in the pupal chamber for 6–17 days (n = 58) before it emerged from the sand. It was dark-brown in color and began to court and mate. The teneral adult was able to survive for at least 20 days without food. There were no significant differences in body parameters between teneral and mature adults (Table 6), except body weight as the ternal adult was significantly lower in weight than the mature adult (t_216_ = 6.08, P < 0.0001). The teneral adult period lasted 10.12 ± 2.46 days, and viability was 93.59% (n = 78).

## Discussion

*Microdera punctipennis* adults were collected from Fukang, the southern fringe of the Gurbantonggut desert, which is about 100 km north of the geological center of Asia. *M. punctipennis* is a unique species in the Gurbantonggut desert (Huang et al. 2005). It adopts several strategies to survive desert environmental extremes. *M. punctipennis* has become nocturnal thereby avoiding the excessive heat and low humidity of their environment; they burrowed in sand or the Substrate around roots of shrubs during the day. In dusk, *M. punctipennis* began to feed, mate, and oviposit. This is a primary response of desert tenebriond beetles to heat and dryness (Wharton 1983). *M. punctipennis* has evolved the flattened body and short legs that are suitable for burrowing into sand, and a fused subelytral cavity for body water conservation, which are the typical morphological features of desert beetles (Draney 1993). The homogeneous and compact eggshell and the sticky layer on the egg surface may be other adaptations for desiccation resistance. The researchers also found that the female kicked sand to bury eggs after oviposition. In addition, *M. punctipennis* larvae have developed morphological characters for sand living, such as sharp and strong chitinized prothoracic legs, pygopods, a hard cephalic capsule, and flat body shape. Active absorption of atmospheric water vapor through rectum has been found in *Tenebrio molitor* larvae (Coutchié and Machin, 1984). On the contrary, *M. punctipennis* prepupa anus was plugged with meconium, which may function in the protection of body water from loss.

**Table 5.**
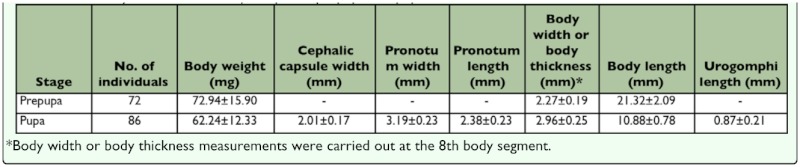
The body dimensions of *M. punctipennis* prepupa and pupa reared at 30±0.5°C, 30± 6% RH and 16L:8D.

**Table 6.**
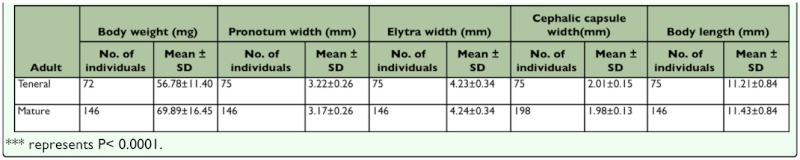
Body dimension and body weight of laboratory reared teneral and mature adults.

Seven instars in total were identified in *M. punctipennis* under the rearing conditions both by the number of molts and frequency distribution of larval body parameters. The later method was used in the instar determination in other insects (Bailez et al. 2003, Maria Do Rosário, 2007; Panzavolta, 2007). It was reported that Tenebrionid beetles *Sternoplax setosa* had 6–8 instars (Zhang et al. 2005a), *Platyscelis hauseri* had 9 instars (Yu et al. 2000), *Blaps femoralis* had 11 instarts (Yu and Zhang 2005), and *Blaps kiritshenkoi* had 12 instars (Zhang et al. 2005b). Draney (1993) reported that for all known desert tenebrionids the number of instars often exceeds 10, influenced by intrinsic and extrinsic factors, while Cloudsley- Thompson (2001) suggested that a reduced number of instars is an adaptation for desert beetles to their arid environment. Further investigations is needed to obtain life cycle data for this beetle in its natural environment. The influence of environmental factors on the number of instars and the duration of each instar need to be elucidated.

The duration of each instar under the rearing conditions could be predicted by the function of Y = 2.25 × -0.83, indicating that the duration in each successive instar was 2.25 days. The overall larval stage from the 1^st^ to the 7^th^ instar lasted about 56 days. This rate of development was greatly reduced compared to other desert tenebrionids (Zhang et al. 2004; Yu and Zhang 2005; Yu and Zhang 2004) reared under uncontrolled laboratory conditions. The reduced duration time in the rearing system described in the present work may suggest a profound influence of temperature on larva development.

The *M. punctipennis* pupa was initially creamy white, which was different from the brownish-yellow pupa of *Dasylepida* sp. (Coleoptera: Scarabaeidae) (Oyafuso et al. 2002). The newly eclosed *M. punctipennis* adult was similar to the teneral adult of beetle *Luprops tristis* (in Tenebrionidae) in that both were inactive until the body color turned dark (Sabu et al. 2008). But the just emerged *M. punctipennis* adult was white, instead of the brown color of *Luprops tristis* and *Librodor japonicus* (Coleoptera: Nitidulidae) (Okada and Miyatske 2007). The adult *M. punctipennis* became brown 48 hours after eclosion,.

The main problem in *M. punctipennis* rearing is the larval cannibalism, and it can be overcome by rearing one larva per bottle, which also provides a convenient way to observe the development of successive larval instars. Sabu et al. (2008) reported a similar rearing method for beetle *Luprops tristis.* In addition, the method discussed in this work provides a way to control sand moisture, which was a key factor for *M. punctipennis* (Bailez et al. 2003). This method also was effective in rearing other desert beetles in the laboratory. There are huge changes from egg to larva to pupa and the mature adult. The establishment of the rearing method not only provides a complete system for observing the biology of this beetle, but also the possibility for investigating these changes using molecular techniques.
